# The complete mitochondrial genome of *Echiniscus testudo* (Heterotardigrada: Echiniscidae)

**DOI:** 10.1080/23802359.2018.1495118

**Published:** 2018-08-01

**Authors:** Kazuharu Arakawa

**Affiliations:** a Institute for Advanced Biosciences, Keio University, Tsuruoka, Japan;; b Faculty of Environment and Information Studies, Keio University, Fujisawa, Japan

**Keywords:** Mitochondrial genome, *Echiniscus testudo*, tardigrade, PacBio sequencing

## Abstract

The complete mitchondrial genome of *Echiniscus testudo*, a cosmopolitan heterotardigrade collected in Japan, has been sequenced using a single molecule real-time (SMRT) sequencing long read after whole genome amplification from a single individual. The genome has a total length of 15,817 bp, consisting of 13 protein-coding genes, 20 tRNA, 2 rRNA genes, and an AT-rich control region. The nucleotide composition was extremely AT-rich, with 78.41% AT. Being in a different class of the phylum Tardigrada, there is a large sequence divergence from the previously reported mitochondrial genome of the eutardigrade *Ramazzottius varieornatus*, but the gene order of protein-coding genes are mostly conserved. This is the first report of a complete mitochondrial genome of a heterotardigrade.

Tardigrades are meiofaunal ecdysozoans with about 1200 species described to date. Of the two classes of tardigrades, heterotardigrades remains mostly unexplored through molecular studies, mostly due to the difficulty for sustainable culture in labs. Two genome sequences have been reported in eutardigrades (Arakawa et al. 2016; Hashimoto et al. 2016), but only an EST study have been reported thus far for heterotardigrades (Forster et al. 2009). Phylogenetic placement of Tardigrada within ecdysozoans still remains unsolved (Yoshida et al. 2017), and molecular studies of heterotardigrades would be a key data for this purpose. In this regard, here we present a complete mitochondrial genome sequence of a heterotardigrade *Echiniscus testudo*.

Single individual of *E. testudo* was isolated from a moss sample collected in Tsuruoka City, Japan (38.739641, 139.807600). The specimen is stored in the Institute for Advanced Biosciences, Keio University, Japan. After thoroughly washing the tardigrade to remove any remaining contaminants, high molecular weight DNA was extracted using MagAttract HMW DNA Kit (QIAGEN) and was amplified using Repli-G Midi Kit (QIAGEN). Purified DNA was then sequenced using PacBio RSII at Takara Bio. Longest read matching to mitochondrial genes was selected using BLAST searches (Altschul et al. 1997), and the read was subsequently error corrected with Illumina reads using Pilon (Walker et al. 2014). Circularity was checked manually, and the genome was annotated using MITOS2 WebServer (Bernt et al. 2013).

The complete mitochondrial genome sequence of *E. testudo* has a total length of 15,817 bp DDBJ accession number LC385650), consisting of 13 protein-coding genes, 22 tRNA, 2 rRNA genes, and an AT-rich control region. Because *E. testudo* belongs to a different class from previously sequenced tardigrade mitogenomes (*Ramazzottius varieornatus* and *Hypsibius dujardini*), sequence is highly diverged from these two genomes. On the other hand, gene order of protein-coding genes is surprisingly conserved. Phylogenetic analysis of the mitochondrial genomes of Tardigrada, Nematoda, and Arthropoda with Priapulida as an outgroup positions Tardigrada close to nematodes (Figure 1). This is in line with previous phylogenetic studies using coding genes and provides a new perspective on the position of tardigrades within ecdysozoans.

**Figure 1.**
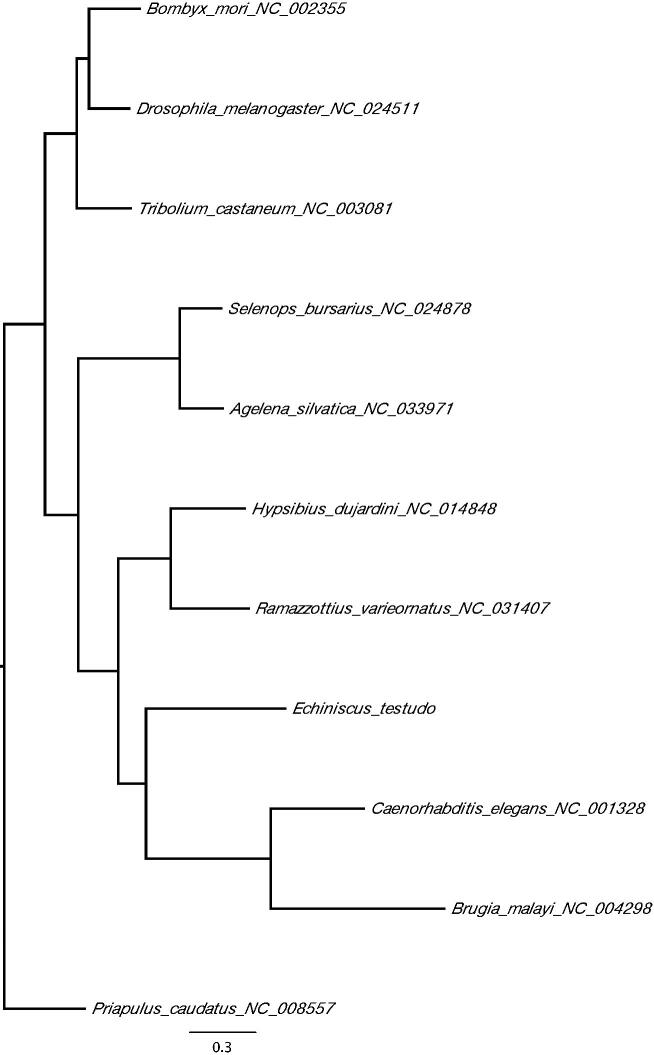
A maximum-likelihood tree of the phylogenetic position of *E. testudo* among other ecdysozoan species. The tree was calculated from concatenated amino acid sequences of 13 mitochondrial protein genes using multiple alignment with MAFFT (Katoh and Standley 2014), followed by Trimal (80% consensus) and Fasttree (Price et al. 2009). Tree is visualized with FigTree (http://tree.bio.ed.ac.uk/software/figtree/). *Priapulus caudatus* was used as an outgroup. GenBank accession numbers of mitogenome sequences used is shown after the species names.
